# Evaluation of the safety profile and therapeutic efficacy of remdesivir in children with SARS-CoV-2 infection – a single-center, retrospective, cohort study

**DOI:** 10.1007/s00431-023-05287-4

**Published:** 2023-10-21

**Authors:** Karolina Kautsch, Joanna Wiśniowska, Joanna Friedman-Gruszczyńska, Piotr Buda

**Affiliations:** 1 Department of Pediatrics, Nutrition and Metabolic Disorders, Children’s Memorial Health Institute, Warsaw, Poland; 2 Department of Cardiothoracic Surgery, Children’s Memorial Health Institute, Warsaw, Poland

**Keywords:** COVID-19, Pediatric population, Remdesivir, SARS-CoV-2

## Abstract

Despite 3.5 years of the SARS-CoV-2 pandemic, we still lack effective drugs against COVID-19. The first and most widely used drug, remdesivir, has not yet been shown to be effective in adults. Even less is known about its effectiveness in children. Therefore, the aim of this retrospective study was to evaluate the safety and efficacy of remdesivir in pediatric patients with COVID-19 hospitalized in one medical center. The medical records of 328 children with COVID-19 were analyzed. Analysis was performed on the subgroups of children treated and not treated with remdesivir. Clinical data on general health, course of COVID-19 and treatment received were analyzed. Remdesivir was administered to 64 children, 16 to treat severe or critical illness and 48 because of the presence of risk factors to prevent progression to severe COVID-19. In children with severe COVID-19, remdesivir did not reduce the mortality rate. However, in patients with milder disease and risk factors, the drug significantly reduced the risk of progression to severe disease. Among adverse events, only mild aminotransferase elevations were observed in 4 patients, but none of these required discontinuation of treatment.

*Conclusions*: Remdesivir is a safe treatment option for children with COVID-19. However, the efficacy of this therapy is still uncertain. It appears that in children with asymptomatic to moderate COVID-19 and risk factors for severe disease, remdesivir could be an effective method of prophylaxis. However, its efficacy in controlling severe disease is questionable and requires further study.

**Table:** 

**What is Known:** *• There are still no effective drugs to combat COVID-19, and the efficacy of the widely used remdesivir in adults is controversial.* *• All recommendations and guidelines on the use of remdesivir in the pediatric population are based mainly on clinical trials in adults.*	
**What is New:** *• Remdesivir is a safe treatment for COVID-19 in the pediatric population.* *• In children with asymptomatic to moderate COVID-19 and risk factors for severe disease, remdesivir could be an effective drug to prevent disease progression. However, its efficacy in treating severe disease in children needs further exploration.*

**What is Known:**

*• There are still no effective drugs to combat COVID-19, and the efficacy of the widely used remdesivir in adults is controversial.*

*• All recommendations and guidelines on the use of remdesivir in the pediatric population are based mainly on clinical trials in adults.*

**What is New:**

*• Remdesivir is a safe treatment for COVID-19 in the pediatric population.*

*• In children with asymptomatic to moderate COVID-19 and risk factors for severe disease, remdesivir could be an effective drug to prevent disease progression. However, its efficacy in treating severe disease in children needs further exploration.*

## Introduction

Coronavirus disease 2019 (COVID-19) associated with severe acute respiratory syndrome coronavirus 2 (SARS-CoV-2) has become one of the most challenging global public health emergencies in recent decades. On March 11, 2020, the World Health Organization (WHO) made the official assessment that COVID-19 can be characterized as a pandemic [1]. International efforts have been launched to develop both a safe and effective vaccine and appropriate therapeutic strategies [2]. Progress in understanding the pathogenesis of SARS-CoV-2 infection has led to the introduction of several therapeutic options, particularly in the adult population, who are at higher risk of developing severe pneumonia and other complications, including death [2, 3]. Remdesivir was the first such drug to be approved in Europe (July 3, 2020) [4].

Remdesivir is an adenine nucleotide analog that is metabolized in cells to the active form, remdesivir triphosphate. Then, as an analog of adenosine triphosphate, it selectively inhibits the RNA polymerase of several viruses, including SARS-CoV-2. Remdesivir is most effective in the first five days of infection when viral load is high [4, 5]. It was developed in 2013 during work on a drug against hepatitis C virus (HCV), and then its effectiveness against viruses such as respiratory syncytial virus (RSV), severe acute respiratory syndrome coronavirus (SARS-CoV) and Middle East respiratory syndrome coronavirus (MERS-CoV) was observed [6]. It was developed in 2017 for the treatment of Ebola virus infection, where it did not achieve therapeutic efficacy, but its safety profile allowed the initiation of drug repurposing research, including SARS-CoV-2 [3, 5].

Remdesivir was approved by the Food and Drug Administration (FDA) on October 22, 2020 and by the European Medicines Agency (EMA) on July 3, 2020 for the treatment of SARS-CoV-2 [4, 5, 7]. The first therapeutic indications were established for patients 12 years of age or older and weighing at least 40 kg. Then on April 25, 2022 (by the FDA) and on September 16, 2020 (by the EMA) remdesivir has become the first drug approved in COVID-19 treatment for children 28 days of age or older and weighing at least 3 kg, who are either hospitalized or non-hospitalized and at high risk for progression to severe COVID-19 [2, 4, 5, 7, 8].

Although differences in the immune response contribute to a less complicated course of disease in children than in adults, this does not mean that severe disease does not occur in children. Furthermore, there are groups at increased risk of severe COVID-19, including children with immunodeficiencies [9–11]. They need both a safe and effective method of COVID-19 treatment and prophylaxis against SARS-CoV-2 infection.

To date, only a few clinical practice guidelines have been developed for children with COVID-19. This is partly due to the very limited number of pediatric clinical trials of drugs used to treat COVID-19 [3, 12]. Remdesivir was not only the first drug approved for the treatment of COVID-19 but also the first and most widely used drug in children. However, we still do not know how safe and effective it is in the fight against SARS-CoV-2 infection in this group of patients due to the lack of sufficient high-quality studies. The controversies regarding the efficacy of remdesivir in adults make it even more necessary to evaluate the use of this drug in the pediatric population [13].

The aim of this study was to evaluate the safety profile and therapeutic efficacy of remdesivir in children with COVID-19 hospitalized at the Children's Memorial Health Institute in Warsaw, Poland.

## Materials and methods

### Study group

This retrospective cohort study was performed on 328 children diagnosed with COVID-19 who were hospitalized at the Children's Memorial Health Institute in Warsaw, Poland, between September 2020 and February 2023. The diagnosis of COVID-19 was confirmed by nasopharyngeal swabs followed by reverse transcription-polymerase chain reaction (RT-PCR) or rapid antigen tests performed before or during hospitalization.

### Division of the study group

Study participants were stratified into two separate cohorts according to whether or not they had received remdesivir. Indications for treatment with remdesivir, according to Polish recommendations [11], were as follows:

1. children with pneumonia requiring oxygen therapy in the course of COVID-19
2. children who do not require oxygen therapy but have an increased risk of progression to severe COVID-19. These risk factors include obesity, congenital heart disease, metabolic disorders, neurological disease, oncological treatment, congenital immunodeficiency, chronic kidney disease, cystic fibrosis, bronchopulmonary dysplasia, organ transplantation, poorly controlled diabetes, asthma, infancy, and neonatal period.

Although the first indication—according to the summary of product characteristics—is reserved for children aged at least 4 weeks and weighing at least 3 kg, and the second for children aged over 12 years and weighing at least 40 kg, in our study the drug was also administered off-label to younger children (below the age of approval) in both indications, after obtaining written consent from the legal guardian.

However, not every patient who met the above indications received the drug. This was due to reasons such as the pandemic period before remdesivir was approved, unavailability of the drug in the hospital, lack of consent from the legal guardian, or rapid progression and death of the patient soon after hospitalization.

### Clinical and laboratory data

The following data were collected for all patients:

- Clinical data: age, sex, presence of immunodeficiency and other risk factors for severe COVID-19 course, vaccination status; reasons for admission; manifestation of the disease, its course and outcome; time of hospitalization; time of treatment initiation and duration, indications, its tolerability and side effects; time to negative antigen test result (if performed);
- Laboratory data: transaminase activity and serum creatinine levels measured before, during and after treatment with remdesivir.

*Course of SARS‑CoV‑2 infection* [14]

- Asymptomatic – positive test for SARS-CoV-2 with no symptoms of COVID-19
- Mild – various signs and symptoms of COVID-19 (e.g., fever, cough, sore throat, headache, muscle pain, vomiting, diarrhea, loss of taste and smell) but no signs of lower respiratory tract infection (e.g. shortness of breath, dyspnea, or abnormal chest imaging)
- Moderate – evidence of lower respiratory tract infection during clinical assessment or imaging with an oxygen saturation (SpO2) ≥ 94% on room air at sea level
- Severe – SpO2 < 94% on room air at sea level, tachypnoea, or lung infiltrates > 50% in chest imaging
- Critical – respiratory failure, septic shock, and/or multiple organ dysfunction

### The use of remdesivir

Remdesivir was administered as follows: on the first day of treatment at a dose of 5 mg/kg (max 200 mg) and on subsequent days at a dose of 2.5 mg/kg (max 100 mg) in a single intravenous infusion. The duration of treatment ranged from 3 to 10 days, depending on the indication and the patient's general condition. Drug administration was started as soon as possible after the indication for treatment was established, as the drug has been shown to be most effective during the viral replication period, up to 7 days after the onset of symptoms. Blood creatinine levels and transaminase activities were always measured before starting treatment.

The following measurements were adopted as contraindications to treatment:

1. alanine aminotransferase (ALT) activity > 5 times the upper limit of normal (ULN) for age
2. estimated glomerular filtration rate (eGFR) < 30 ml/min

The following contraindications have been adopted for remdesivir continuation therapy:

1. increase in ALT ≥ 5 times ULN during treatment
2. any increase in ALT associated with signs or symptoms of hepatitis
3. decrease in eGFR < 30ml/min during treatment

### Statistical analysis

All data were analyzed using Microsoft Excel, R and Statistica 12 software. The normality of quantitative data was assessed using Shapiro–Wilk test. If their distribution differed significantly from a Gaussian distribution, the Wilcoxon matched pairs test was used for dependent data and the Mann–Whitney U test for independent data. Qualitative data were compared using the chi-squared test or its modifications. A probability value of p < 0.05 was considered statistically significant.

### Ethical considerations

The study was conducted in accordance with the Declaration of Helsinki and approved by the institutional ethics committee of the Children's Memorial Health Institute in Warsaw, Poland (No. 34/KBE/2023).

## Results

### Characteristics of the group

The median age of the study group (141 girls and 187 boys) was 4 years but ranged from 1 week to 18 years. Children with risk factors for severe COVID-19 accounted for 78.96% (n = 259) of the study population, while those with immunodeficiency accounted for 35.98% (n = 118). Detailed risk factors for severe COVID-19 are shown in Table 1. Only 4 patients (1.22%) were fully vaccinated and 3 (0.91%) were partially vaccinated.

**Table 1:** Risk factors for the severe course of COVID-19 in the studied population

**Risk factors**	**n**	**%**
none	64	22.56%
infancy / neonatal period	98	29.88%
oncological treatment	72	21.95%
neurological diseases	38	11.59%
chronic cardiac defects	25	7.62%
solid organ transplantation	20	6.10%
primary immune disorders	15	4.57%
obesity	8	2.44%
pulmonary diseases	7	2.13%
chronic kidney disease	7	2.13%
inflammatory bowel disease	6	1.83%
autoimmune diseases	5	1.52%
metabolic diseases	3	0.91%
diabetes	3	0.91%
other	3	0.91%

### Course of COVID-19

The course of COVID-19 was predominantly asymptomatic (n = 106; 32.32%) or mild (n = 170; 51.83%), while more severe courses were observed significantly less frequently (Table 2). 48 children (14.63%) developed pneumonia and 37 of them required oxygen therapy, mostly passive (n = 21), in 5 cases high flow and in 6 cases mechanical ventilation. Only 9 patients (2.74%) needed hospitalization in the intensive care unit (ICU) and 5 died (1.52%). The median length of hospital stay was 8 days (ranging from 1 to 123 days).

**Table 2:** Comparison of children treated and not treated with remdesivir

		**entire study group (n = 328)**	**treated with remdesivir (n = 64; 19.51%)**	**not treated with remdesivir (n = 264; 80.49%)**	***p***
**age, y, median (range)**		4 (0.7–11)	3.8 (0.8–10.3)	4 (0.7–11)	0.58
**sex, girls:boys**		141: 187	26: 38	115: 149	0.66
**immunodeficiency**		118 (35.98%)	28 (43.75%)	88 (33.33%)	0.12
**vaccination (any doses)**		7 (2.1%)	0	7 (2.65%)	0.67
**course of COVID-19**					
	**asymptomatic**	106 (32.32%)	10 (15.63%)	96 (36.36%)	< *0.01*
	**mild**	170 (51.83%)	24 (37.50%)	146 (55.30%)	
	**moderate**	22 (6.71%)	13 (20.31%)	9 (3.41%)	
	**severe**	23 (7.01%)	13 (20.31%)	10 (3.79%)	
	**critical**	7 (2.13%)	4 (6.25%)	3 (1.14%)	
**adverse outcomes**					
	**pneumonia**	48 (14.63%)	24 (37.50%)	24 (9.10%)	< *0.01*
	**oxygen therapy**	39 (11.89%)	22 (34.38%)	17 (6.44%)	< *0.01*
	**hospitalization in the ICU**	9 (2.74%)	5 (7.82%)	4 (1.52%)	*0.01*
	**death**	5 (1.52%)	3 (4.69%)	2 (0.76%)	*0.02*
**symptoms**					
	**fever**	167 (50.91%)	43 (67.19%)	124 (46.97%)	< *0.01*
	**cough**	94 (28.66%)	31 (48.44%)	63 (23.86%)	< *0.01*
	**dyspnea**	42 (12.80%)	22 (34.38%)	20 (7.58%)	< *0.01*
	**vomiting**	35 (10.67%)	8 (12.5%)	27 (10.23%)	0.66
	**diarrhea**	27 (8.23%)	3 (4.69%)	24 (9.10%)	0.24
**time of hospitalization, days, median (range)**		8 (4–13)	12 (7–15)	7 (4–11)	< *0.01*
**time to negative antigen test result, days, median (range)**		9 (12–16)	7 (11–14)	10 (13–18)	0.08

Italic entries reflect statistical significance (*p *< 0.05)

*COVID-19* Coronavirus Disease 2019, *ICU* intensive care unit

### Use of remdesivir

In the study population, 64 children (19.51%) received remdesivir. The median duration of treatment was 5 days (range 3–10 days). The drug was most commonly started on the second day of treatment (n = 14; 23.33%). Remdesivir was administered mainly due to the presence of risk factors for a severe course of COVID-19 (48 patients; 75.00%) and due to a severe course of the disease with the need for oxygen therapy (n = 16; 25.00%). A comparison of patients treated and not treated with remdesivir is shown in Table 2.

### Safety

**Fig. 1:**
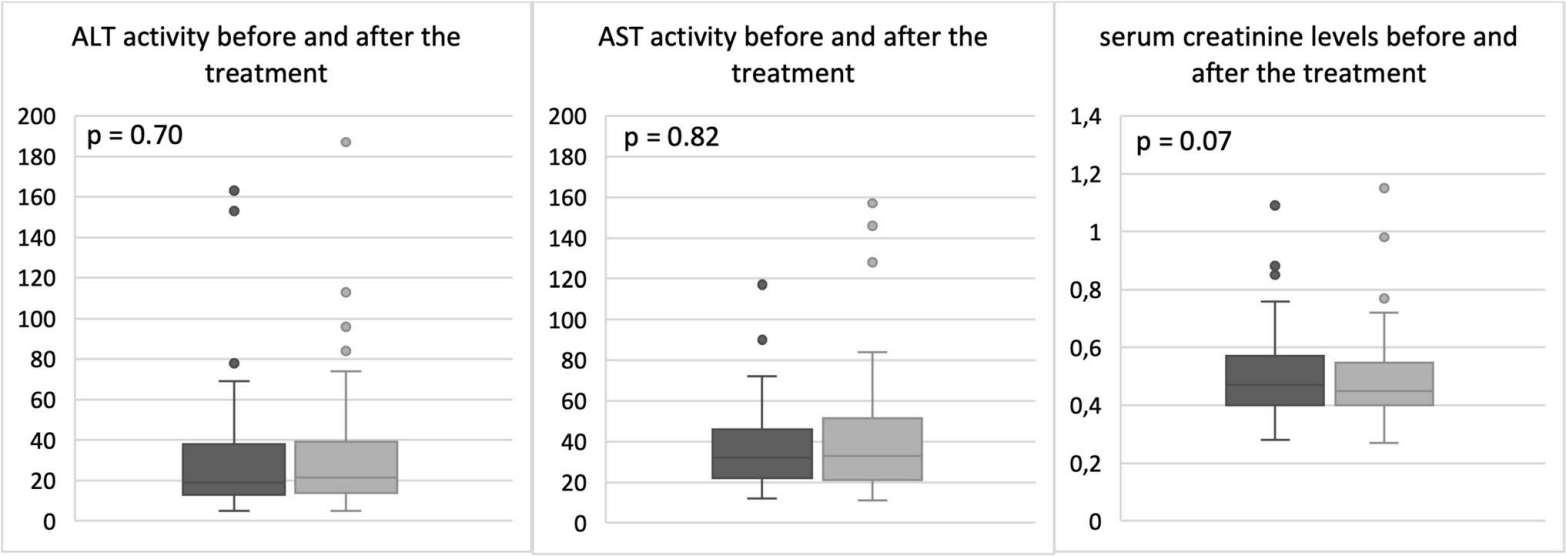
Comparison of transaminases activity and serum creatinine levels in the study group before and after the treatment with remdesivir

We observed a moderate increase in transaminase activity (max. 3 times ULN) in 4 patients, but due to the insignificance of the increase and the absence of hepatitis symptoms, none of them had to discontinue therapy. The values of aminotransferase activity before and after treatment with remdesivir are shown in Fig. 1, and the difference between them was not statistically significant. We also did not observe any other side effects of the drug, including renal failure (Fig. 1) or bradycardia, as described in the literature.

### Efficacy

In terms of treatment effect, of the patients who received remdesivir due to a severe or critical COVID-19 course, 13 (81.25%) made a full recovery, and 3 (18.75%) had no treatment effect, resulting in death. However, children not treated with remdesivir had comparable clinical outcomes (Table 3).

**Table 3:** Comparison of the clinical effect in patients treated and not treated with remdesivir depending on the indications and course of COVID-19

**children with severe / critical course of COVID-19**			
**clinical effect**	**treated with remdesivir** **(n = 16, 55.17%)**	**not treated with remdesivir** **(n = 13; 44.83%)**	***p***
full recovery	13 (81.25%)	11 (84.62%)	0.69
death	3 (18.75%)	2 (15.38%)	0.69

**children with initially asymptomatic to moderate COVID-19 and risk factor for severe disease**			
**final course of COVID-19**	**remdesivir in prophylaxis** **(n = 48; 19.35%)**	**no remdesivir in prophylaxis** **(n = 200; 80.65%)**	***p***
asymptomatic	10 (20.83%)	68 (34.0%)	< *0.01*
mild	23 (47.92%)	112 (56.0%)	
moderate	15 (31.25%)	7 (3.5%)	
severe	0 (0.0%)	10 (5.0%)	
critical	0 (0.0%)	3 (1.5%)	
death	0 (0.0%)	2 (1.0%)	*0.04*

**children with initially asymptomatic to moderate COVID-19 and immunodeficiency**			
**final course of COVID-19**	**remdesivir in prophylaxis** **(n = 24; 21.05%)**	**no remdesivir in prophylaxis** **(n = 90; 78.95%)**	***p***
asymptomatic	9 (37.50%)	43 (47.78%)	*0.04*
mild	11 (45.83%)	44 (48.89%)	
moderate	4 (16.67%)	2 (2.22%)	
severe	0 (0.0%)	0 (0.0%)	
critical	0 (0.0%)	1 (1.11%)	
death	0 (0.0%)	1 (1.11%)	0.60

Italic entries reflect statistical significance (*p *< 0.05)

Of the 264 children with risk factors for severe COVID-19, 15 received remdesivir because of their initial severe general condition (5.68%) and 48 prophylactically (18.19%), while 200 (75.76%) were treated only symptomatically. Table 3 compares the course of disease in children with risk factors who received prophylaxis with remdesivir and those who did not. No progression to severe or critical illness or deaths were observed in the prophylaxis group. Among patients with risk factors, a subgroup with immunodeficiencies was also identified, in whom the benefit of prophylactic use of the drug was also demonstrated.

## Discussion

Although remdesivir is widely used in children, studies evaluating its safety and efficacy in this age group are still lacking. Any recommendations regarding the use of remdesivir in the pediatric population are mainly based on clinical trials in adults. To date, only the first results from an open-label phase 2/3 clinical trial—the CARAVAN study—have been published. The authors focused mainly on the safety evaluation of remdesivir in COVID-19 treatment in children [7]. According to this study, which involved 54 patients, the drug was safe and well tolerated in children. The most common adverse events were constipation (17% of patients) and acute kidney injury (11%). Other adverse events included increased ALT activity, hyperglycemia and bradycardia.

There are several other single-center observational studies in the literature evaluating the safety of remdesivir in children. However, all of these studies are based on small groups of patients. In a study by Samuel et al., 48 children received remdesivir, but only 22 received the full course of treatment [8]. Approximately 20% of patients experienced bradycardia and hypertension during drug administration, but these did not require discontinuation of therapy. No drug-induced liver or kidney damage was observed in any of the patients. In another case series from Japan, remdesivir was administered to 20 children with a satisfactory clinical outcome and no serious adverse events [15]. Mild adverse events included elevated liver enzymes in 4 children, leukopenia in one child and neutropenia in another. In a study describing the use of remdesivir in children with severe COVID-19, 32% of patients experienced at least one adverse event during treatment, the most common of which were elevated transaminase levels and renal adverse events. In five patients, remdesivir had to be discontinued due to elevated liver enzyme levels, rash or relapse of acute lymphoblastic leukemia [16]. As the active metabolite of remdesivir accumulates in the kidneys, liver and gastrointestinal tract, it seems obvious that the most common adverse effects are renal injury and liver enzyme elevation [5]. Although we observed a moderate increase in transaminase activity in a few patients, none of them required discontinuation of therapy or showed signs of hepatitis. We also cannot exclude the possibility that transaminase elevation was related to hepatitis due to SARS-CoV-2 infection. We did not observe any renal side effects or hypersensitivity reactions.

There have also been anecdotal reports of remdesivir-induced bradycardia in children. Some investigators, based on their experience, suggest the need for cardiovascular monitoring during remdesivir therapy as significant bradycardia may occur in treated patients [17, 18]. At present, the mechanism by which remdesivir causes bradycardia is not fully understood. The most widely speculated explanation is that the drug, as an adenosine analog, may block the atrioventricular node or bind to human mitochondrial RNA polymerase and cause cardiotoxicity [5]. However, we did not observe any adverse cardiovascular symptoms in our patients, and all were carefully monitored during the acute phase of the disease.

Although there are reports on the safety of remdesivir and its relatively good tolerability in the pediatric population, there is still a lack of reliable studies evaluating the efficacy of this treatment. This is particularly important in light of recent reports based on studies in the adult population showing that remdesivir most likely does not reduce the risk of death or adverse events in COVID-19 [13]. Almost all of the studies cited above, which showed that the majority of children treated with remdesivir recovered, lacked a control group [7, 8, 15, 16]. In the CARAVAN study, 85% of patients achieved clinical improvement during treatment, leading to the conclusion that remdesivir is a treatment option for pediatric patients with COVID-19 [7]. However, in the absence of a control group, such firm conclusions cannot be drawn. Only Shoji et al. planned a retrospective cohort study with a propensity score-matched control group. They found only one statistically insignificant difference between the groups. A higher percentage of children treated with remdesivir achieved defervescence on day 4. In addition, there was no apparent clinical efficacy of remdesivir in hospitalized children with mild to moderate COVID-19 [19]. To our knowledge, no one has thus far compared the effect of COVID-19 treatment with and without the use of remdesivir in severe or critical illness in children. In our analysis, remdesivir did not have a statistically significant benefit in improving survival in children with severe or critical COVID-19. However, this analysis was performed on a small study group, so it is difficult to draw firm conclusions.

Another important aspect of the effectiveness of remdesivir is the prevention of severe COVID-19 in patients with risk factors. The PINETREE trial found that a three-day course of remdesivir was associated with a reduced risk of hospitalization and death in adults with risk factors and mild COVID-19 [20]. To our knowledge, thus far, only one study has evaluated the use of remdesivir in children with risk factors for severe COVID-19. Researchers from Singapore reported that in their 4 oncology patients treated with remdesivir, they found no clear benefit in terms of time to viral clearance. However, they did not assess the risk of progression to severe COVID-19 [21]. We have shown that patients with risk factors for severe COVID-19 have a significantly lower risk of disease progression after receiving remdesivir prophylaxis. To our knowledge, this is the first study to address this issue in children using such a large group of patients.

Our study has several limitations. The most important is the small size of the study group treated with remdesivir. However, to the best of our knowledge, it is still one of the largest pediatric populations described in the literature where this treatment has been administered and who were burdened with so many and diverse risk factors for progression to severe COVID-19. Another limitation is the retrospective nature of the study and the lack of randomization. Patients' recovery or lack of progression to severe disease could have been due to many other factors. The outcome of COVID-19 depends to a large extent on the response orchestrated by the immune system, which is still not fully understood and predictable, and on which the medical staff still has little influence.

## Conclusions

In conclusion, remdesivir is a safe form of treatment for COVID-19, with a small number of side effects that in the vast majority of cases do not require discontinuation of treatment; yet, the effectiveness of this therapy is still uncertain. In children with asymptomatic to moderate COVID-19 who have risk factors for severe disease, remdesivir appears to be an effective prophylactic treatment. However, its efficacy in controlling severe and critical forms of the disease is questionable and requires further prospective and randomized studies in a larger group of pediatric patients.

## Data Availability

The datasets analyzed during the current study are available from the corresponding author on reasonable request.

## Abbreviations

ALT: Alanine aminotransferase
COVID-19: Coronavirus disease 2019
eGFR: Estimated glomerular filtration rate
EMA: European Medicines Agency
FDA: Food and Drug Administration
HCV: Hepatitis C virus
ICU: Intensive Care Unit
MERS: Middle East respiratory syndrome coronavirus
RSV: Respiratory syncytial virus
RT-PCR: Reverse transcription polymerase chain reaction
SARS-CoV: Severe acute respiratory syndrome coronavirus
SARS-CoV-2: Severe acute respiratory syndrome coronavirus 2
SpO2: Oxygen saturation
ULN: Upper limit of normal
WHO: World Health Organization

## Declarations

### Data access

The datasets analyzed during the current study are available from the corresponding author on reasonable request.
